# Instrumentation enabling study of plant physiological response to elevated night temperature

**DOI:** 10.1186/1746-4811-5-7

**Published:** 2009-06-11

**Authors:** Abdul R Mohammed, Lee Tarpley

**Affiliations:** 1 Texas AgriLife Research and Extension Center, 1509 Aggie Drive, Beaumont, Texas-77713, USA

## Abstract

**Background:**

Global climate warming can affect functioning of crops and plants in the natural environment. In order to study the effects of global warming, a method for applying a controlled heating treatment to plant canopies in the open field or in the greenhouse is needed that can accept either square wave application of elevated temperature or a complex prescribed diurnal or seasonal temperature regime. The current options are limited in their accuracy, precision, reliability, mobility or cost and scalability.

**Results:**

The described system uses overhead infrared heaters that are relatively inexpensive and are accurate and precise in rapidly controlling the temperature. Remote computer-based data acquisition and control via the internet provides the ability to use complex temperature regimes and real-time monitoring. Due to its easy mobility, the heating system can randomly be allotted in the open field or in the greenhouse within the experimental setup. The apparatus has been successfully applied to study the response of rice to high night temperatures. Air temperatures were maintained within the set points ± 0.5°C. The incorporation of the combination of air-situated thermocouples, autotuned proportional integrative derivative temperature controllers and phase angled fired silicon controlled rectifier power controllers provides very fast proportional heating action (i.e. 9 ms time base), which avoids prolonged or intense heating of the plant material.

**Conclusion:**

The described infrared heating system meets the utilitarian requirements of a heating system for plant physiology studies in that the elevated temperature can be accurately, precisely, and reliably controlled with minimal perturbation of other environmental factors.

## Background

Global climate warming can affect functioning of crops and of plants in the natural environment. Increased night temperatures have been implicated in decreased crop yields throughout the world and are predicted to warm more than the daytime temperatures in the future [1]. The effects of high night temperatures are diverse, including, for example, increased coincidence of intervals of unusually high night temperature with sensitive reproductive stages eventually resulting in poor seed set and a decline in vegetative reserves due to increased respiration and alteration in phenology, or, in the case of natural populations, altered quantity and seasonal distribution of reproductive units.

Precise and accurate control of the temperatures of and immediately surrounding small populations of plants is a primary purpose of an apparatus for control of high night temperature, but reliability is also needed to avoid short-term deviations of the tissue temperatures beyond physiologically normal ranges. Short-term deviations of tissue temperature have often been shown to affect plant function, often with effects carried beyond the period of exposure. For example, sublethal heat shock, with short-term tissue temperature increases in the range observed in otherwise well-controlled infrared (IR) heating studies, can induce the synthesis of heat shock proteins and other physiological changes that are important for thermotolerance [2]. Another plant-physiological feature that is easy to inadvertently alter during a plant population warming study is vapor pressure deficit, which can lead to decreased leaf water potential for plants under some growing conditions. Decreased leaf water potential can trigger alterations to plants similar to those observed in sublethal heat shock, for example the synthesis of heat shock proteins and other physiological changes that are important for abiotic stress tolerance [2]. One means to alter the vapor pressure deficit (VPD) is to alter the absolute humidity [3]. Global climactic change models predict that absolute humidity will change with global warming [1] indicating that the ability to maintain absolute humidities while altering temperature would be an additional prerequisite for a heating system designed for study of various plant physiological responses to elevated temperature.

Plant physiological experimentation employs both square-wave manipulation of environmental variables as well as ambient +/- some proportion or degree of the quantity of an environmental factor, e.g. average seasonal temperature + x°C (e.g., [4]) depending on the study objectives, thus the inclusion of computer-based data acquisition and control via the internet is highly desirable to facilitate the study of plant physiological responses to various aspects of high night temperature. The responses by plants and plant populations are multiple, so demand the ability to clearly separate these responses via well-replicated studies, often of fairly subtle temperature changes, thus cost and scalability warrant consideration. The apparatus should avoid unintended effects on the local environment.

Current apparatuses used to study the effects of high night temperatures are limited in ability to carefully control the elevated temperature, conduct replicated study of populations of plants, or minimize perturbation of other environmental factors. Greenhouses, growth chambers, phytotrons, open-top chambers (OTC), and naturally-lit plant growth chambers (known as Soil-Plant-Atmosphere-Research (SPAR) units) are usually used in controlled environmental studies. Greenhouses generally have higher humidity, lower wind speed, and lower light intensity compared to outside. Moreover, greenhouse coverings typically transmit two-thirds to three-fourths of the available sunlight [5]. In artificially lit growth chambers, the temperature is well controlled, however plants are subjected to an artificial light environment. The phytotron has similar light conditions as that of artificially lit growth chambers and also has smaller rooting volumes, which might restrict the partitioning of carbohydrates to roots [6]. The OTC requires a high flow rate of air in and out of the OTC to control the temperature and the humidity [5]. However, many studies have reported higher daytime and night temperatures in OTCs compared to neighboring unenclosed areas [7,8]. The SPAR units are one of the best in controlling the environmental factors [4], however the cost and lack of mobility of the units makes them site-specific. In most of the above-mentioned facilities, the climatic conditions are unrealistic and fail to couple changes in light, temperature and other factors resulting in poor simulation of natural environmental conditions [9]. In contrast, the use of an IR heating system can be employed in ways that do not alter other natural environmental conditions such as light intensity, humidity, and wind speed, while precisely controlling temperature.

The use of IR heating for study of plant – and ecosystem response to global warming has been increasing during the last 10 to 15 years. For example, Harte & Shaw (1995) [10] and Harte *et al. *(1995) [11] have conducted a long-term study of the effect of added heat to plots located in a montane community. IR radiation warms the vegetation similar to that of normal solar heating and is energetically efficient because it heats the vegetation directly without having to overcome a boundary layer resistance if the air were to be heated [3]. An improvement in IR heater control was made by Nijs (1996) [12], who varied the heat output in order to maintain a constant 2.5°C difference in canopy temperature compared to the control plots. Free Air Temperature Increase (FATI), as coined by Nijs (1996) [12], is based on modulated IR radiation and increases temperature in a controlled fashioned, without enclosing the plants. More recent reports on the use of IR heating systems for ecosystem warming include Luo et al. (2001) [13], Shaw et al. (2002) [14], Wan et al. (2002) [15], Noormets et al. (2004) [16] and Kimball et al. (2008) [17]. All the above mentioned studies have primarily used IR heating to study the effect of warming at the plant population level, mostly with the intent to estimate possible ecosystem effects of global warming. In contrast, we sought to develop an IR-based system allowing the study of plant physiological responses to high temperature. The purposes of this paper are to explicitly describe the controlling capabilities of the presented IR heating system, provide results indicating that the described apparatus meets the criteria indicated above for use in study of plant physiological response, and to provide additional example results to further characterize the system and illustrate successful application of the apparatus in study of plant physiological response to high night temperature.

## Results and discussion

Trade names and company names are included for the benefit of the reader and do not imply any endorsement or preferential treatment by the authors or Texas AgriLife Research.

### Infrared (IR) heaters

The IR heaters, purchased from Omega (RAD 3113 BV/208, OMEGA Engineering, Inc. Stamford, Connecticut, USA) are housed in a rigid aluminum housing which is 77.8 cm in length and 9.4 cm in width. The aluminum housing is equipped with interlocking connectors, mounting clamp, conduit connector, polished aluminum reflector, and single radiant (RAD) elements. The single RAD element is a rod-shaped heating element (1 cm diameter and 57.8 cm long) mounted at the focal point of the polished aluminum reflector. The working voltage of the heating element is 120 volts and has a power of 1100 watts. The Incoloy (an iron-nickel alloy) sheath is 9.5 mm diameter. The operating wavelengths of the IR heaters are >1200 nm, and the IR heater output is negligible <1200 nm [18]. Hence, there is no significant emission of photo-morphogenic wavelengths. Stranded, insulated, nickel-plated copper wire is used for connecting the heaters to the power controllers. For protection of the IR heaters and personnel, a grill (GR-3, OMEGA Engineering, Inc.) is provided for each IR heater. A detailed description of IR heaters is provided in 'The Electric Heaters Handbook' [18]. Previous studies have described ways to weatherproof similar IR heaters [17].

### Power semi-conductor controllers

A silicon controlled rectifier (SCR) power controller is an output device used for fast heat switching, to control variable resistance heaters and to switch higher amperage electric heaters. In the setup, the IR heaters are controlled using power semi-conductor controllers (SCR71P-208-030-S60, OMEGA Engineering, Inc.) to enable proportioning heating action instead of on/off action. These power controllers are single-phase models, phase-angle firing, 208 volt, 30 amp with a 60-s soft-start option. These power controllers use 'phase-angled fired' proportional control, which eliminates thermal shock and extends the working life of the heating elements. The 'phase-angled fired' proportional SCR control also provides a smooth, rapid (in milliseconds) and controlled heating process. This is potentially advantageous for vegetation warming studies because it provides a nearly continuous adjustment of the heater output thus minimizing the risk of prolonged or intense heating of the plant material with unexpected deviations away from the set point. The power controller receives a 4 – 20 mA process output signal from a temperature controller. This signal is processed by the electronics in the power controller to switch the heaters at sub-cycle intervals, resulting in a smooth radiation output. A detailed description of power semi-conductor controllers is provided in 'The Electric Heaters Handbook' [18].

### i-Series temperature controllers

The basic function of a temperature controller is to compare the actual temperature with the set point temperature and produce an output which will maintain that set point temperature. The i-Series temperature controllers (CNi16D53-C24, OMEGA Engineering, Inc.) used in the present setup include digital panel meters and single loop autotuned proportional integral derivative (PID) control mode controllers that are simple to configure and use, while providing tremendous options including direct connectivity to an Ethernet network with the ability to serve web pages over a Local Area Network (LAN) or the Internet. The i-Series temperature controllers are Deutsche Industrial Norm (DIN) compatible for easy incorporation into industrial mounting and control systems. The instrument utilizes Chip On Board (COB) and Surface Mount Technology (SMT) assembly techniques and automation and provides the ability to program and set the temperatures and alarms. The presented system uses 1/16 DIN Omega-series controllers with dual display, analog output, 0 to 10 Vdc or 0–20 mA, at 500 ohm max, and relay. The embedded internet and serial communication interface allows remote Data Acquisition and Control (DAC) and flexibility in programming via OLE for Process Control (OPC) software on remote PCs. The i-Series temperature controller has both RS-232 and RS-485 serial communication interface, which allows multiple temperature controllers to be connected through a single industrial server. A detailed description and applications of i-Series temperature controllers are provided in 'The Electric Heaters Handbook' [18].

### i-Server

The temperature controllers are connected to an i-Server (EIS-2-RJ, OMEGA Engineering, Inc.) using a RJ45 serial port. The RS-485 interface standard used for connecting the temperature controllers to the i-Server provides distances up to 1200 m, data rates up to 10 Mbps, up to 32 line drivers on the line, and up to 32 line receivers on the same line [19]. The i-Server takes a dynamically assigned Internet Protocol (IP) address from a Dynamic Host Configuration Protocol (DHCP) server on the network. This DHCP client capability is a valuable and unique feature of the i-Server that makes it extremely easy and simple to use on almost any Ethernet network. The i-Server connects to an Ethernet network with a standard RJ45 connector. In addition, the i-Server can be used to create a virtual tunnel on an Ethernet/Internet network simulating a local point-to-point serial connection between a serial device and a PC. The serial devices will function over the Ethernet network or the Internet as if they were connected directly to a PC. The COM port on the i-Server simulates a local COM port on the PC. The i-Series temperature controllers and i-Servers connected to an Ethernet network or Internet makes it possible to monitor and control a process from any remote place. A detailed description of the i-Server and its applications are provided in 'The Electric Heaters Handbook' [18].

### OLE for process control (OPC) server software

The OPC servers are hardware drivers that are written to a common standard, OPC. The OPC compliant programs (OPC Clients) are available for Distributed Control System (DCS), Supervisory Control and Data Acquisition (SCADA) and Human Machine Interface (HMI). Previously each software or application developer was required to write a custom interface to exchange data with hardware field devices. The OPC eliminates this requirement by defining a common, high performance interface. The OPC specification is a non-proprietary technical specification that defines a set of standard interfaces based upon Microsoft's OLE/COM technology. A complete description and specifications of OPC servers and OPC clients are available at the OPC Website [20].

### Thermocouples

The temperature input can be provided through thermocouple, Resistance Temperature Detector (RTD), or process voltage/current. Thermocouples were used in the presented system to provide flexibility in the system, i.e., use of IR (non-contact) thermocouples, rapid response air temperature thermocouples, or hypodermic needle-type (internal temperature of desired plant part) thermocouples as desired. In the presented system, the temperature controllers receive input from the rapid response air thermocouples (GTMQSS-040E-12, OMEGA Engineering, Inc.), which were attached to the temperature controllers by thermocouple wire (304-T-MO-032, OMEGA Engineering, Inc.). The thermocouples are low noise thermocouple probes with type 'T' grounded-junction probe with a Teflon-insulated extension wire and subminiature male connector termination. The thermocouple wire is also a type 'T' wire, MgO insulation, 0.076 cm cable and the sheath material of the wire is 304 stainless steel (Omegaclad thermocouple wire, The Electric Heaters Handbook, Omega, 2008).

### Setup and working of IR heating system

**Figure 1:**
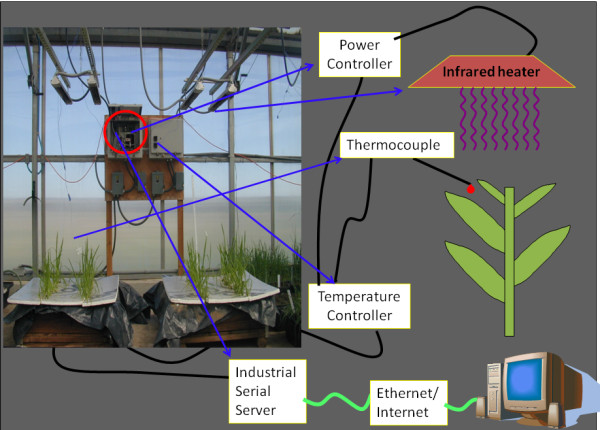
**Picture showing cartoon and actual setup of IR heating system**.

The thermocouple is attached to the i-Series temperature controller by thermocouple wire, which is a type 'T' grounded-junction probe with a Teflon-insulated extension. The i-Series temperature controller also communicates with the power controller and i-Server. The i-Series temperature controller communicates with the power controller by electrical wire connections and with the i-Server through an RS-485 interface via a RJ45 serial port. The power controllers are connected to the IR heaters by stranded, insulated, nickel-plated copper wire. The i-Server communicates with the Ethernet/Internet via a RJ45 serial port. The OLE software is installed on a PC connected via the Ethernet/Internet. The temperature can be set at predetermined set points using i-Series temperature controllers, which can be accessed from a remote distance through a PC via the internet and i-Server. Sophisticated temperature regimes can be applied through use of the OLE software, as can data acquisition. The model of the setup and the actual setup are shown in Fig. 1.

Air temperatures can be set at predetermined set points using i-Series temperature controllers. When the temperature is below the set point as determined by the readings from the thermocouples, a signal from an i-Series temperature controller is sent to a power controller, which in turn controls the heater output to maintain the temperature very near the set point.

### Plant culture and temperature treatments

Three experiments were conducted in the greenhouse at the Texas AgriLife Research and Extension Center at Beaumont, Texas, USA. 'Cocodrie', a common U.S. rice (*Oryza sativa *L.) cultivar of tropical japonica background, was used in all the experiments. The average ambient night temperature during the reproductive period of the rice growing season at the location varied between 26 to 28°C [21]. Hence, the ambient and elevated night temperatures were set at 27 and 32°C (ambient plus 5°C), respectively. This is a large temperature difference relative to most vegetation warming studies and is also a square-wave treatment requiring the maintenance of constant temperature over long periods of time, thus challenged the heating system's ability for accurate, precise, reliable heating without causing plant physiological artifacts.

Plants were grown in 3-L pots that were placed in a square box (0.84 m^2^), 10 pots per box. The boxes were lined with black plastic (thickness = 0.15 mm; FILM-GARD, Minneapolis, Minnesota, USA) that served as a water reservoir. Pots were filled with a clay soil (fine montmorillonite and thermic Entic Pelludert [22]. At 20 days after emergence (DAE), the boxes were filled with water to approximately 3 cm above the top of the soil in each pot. A reflective foam cover (Cellofoam Sheathing/Underlayment, Cellofoam. North America Inc., Conyers, Georgia, USA) was placed over the water surface to prevent direct IR heating of water. A three-way split application of nitrogen was used as described by [23]. Nitrogen was applied in the form of urea and ammonium sulfate, and phosphorus in the form of P_2_0_5_. At planting, urea-N was applied at the rate of 113.5 kg ha^-1 ^along with 45.4 kg ha^-1 ^phosphorus (P_2_0_5_). The second and third nitrogen fertilizations (both 79.5 kg ha^-1 ^nitrogen in the form of ammonium sulfate) were applied 20 DAE and at the panicle-differentiation stage.

Plants were subjected to elevated night temperature through the use of the nearly continuously controlled IR heaters, which were positioned 1.0 m above the topmost part of the plants. This involved controlled heating of small unenclosed areas of the greenhouse. Air temperatures were controlled at predetermined set points (27°C and 32°C). The night temperatures were imposed from 2000 h until 0600 h starting from 20 days after emergence until harvest. There were three experiments presented in the present study. The assignment of heat treatments to greenhouse location was random within each experiment. The greenhouse was maintained at 27°C nighttime temperature and within this, plants of the HNT treatment were subjected to elevated nighttime temperature through the use of nearly continuously controlled (sub-second response) IR heaters. In each experiment, there were four sets (replications) of IR heaters, two IR heaters in each set. The night temperature and humidity were independently monitored using standalone sensor/loggers (HOBOs, Onset Computer Corporation, Bourne, Massachusetts, USA) in both the ambient and the HNT regimes. In each temperature regime, 1 m below the IR heaters, there were four HOBOs, one HOBO per replication. In addition, under the HNT regime, there were two additional HOBOs per replication placed at 0.75 m and 1.25 m below the IR heaters to measure the temperature at different levels below the heaters. The HOBOs were set to record temperature and humidity at 15-minutes interval. Hence, the value for a temperature or humidity for a night (2000–0600) is an average of 40 data points. In experiment-I, plants grown under two sets of IR heaters (randomly selected) were exposed to a wind velocity of 2.2 m s^-1 ^using industrial fans with speed controls (Super Fan, Mobile Air Circulator, Air Vent Inc., Dallas, Texas, USA). The wind speed was measured using a wind speed meter (ADC™•WIND™, The Brunton Company, Riverton, Wyoming, USA). In addition to this main study (consisting of three experiments), a preliminary study (one experiment) was done wherein the HNT was maintained at 32°C using greenhouse heaters. In the preliminary study, temperature and humidity were recorded using HOBOs. The data for humidity under IR vs. greenhouse heating was analyzed using a paired t-test.

### Performance of infrared heating system

There was no difference between the experiments (locations within the greenhouse) for ambient as well as elevated night temperatures measured using the HOBOs.

**Figure 2:**
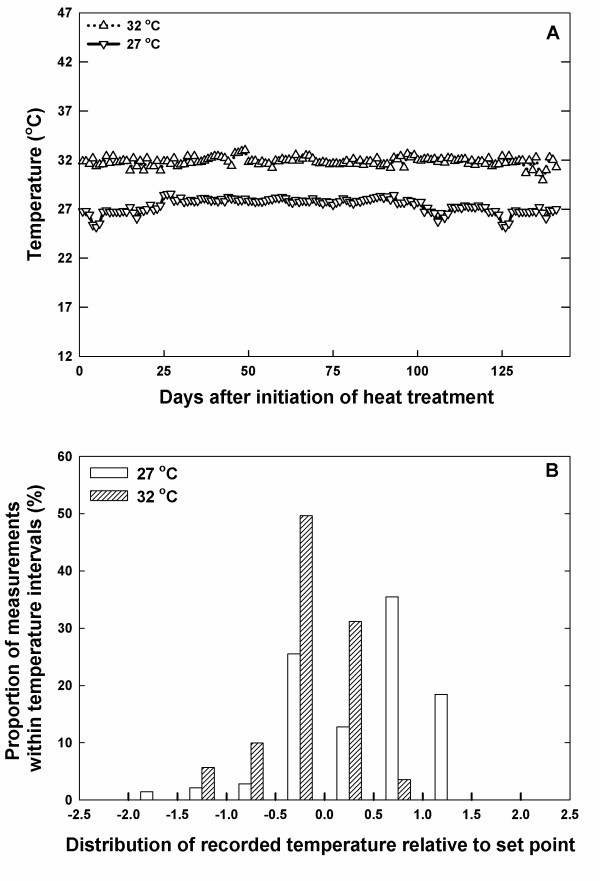
**Ability of IR heating system to maintain temperatures at set points**. The temperatures were monitored using standalone sensor/loggers (HOBOs). The ambient night temperature was set at 27°C and high night temperature at 32°C (A). Distribution of recorded temperature to the set point for ANT and HNT treatments (B).

The IR heaters provided accurate night (2000–0600 h) temperatures during the cropping season (emergence to harvest). The average night temperatures were 27.3 and 31.8°C for the ambient (27°C) and ambient + 5°C (32°C) temperature treatments, respectively (Fig. 2A), indicating the ability of the described apparatus to maintain a large temperature differential for an unenclosed space. For most of the time of heat exposure (82%), night temperature was held within 0.5°C for 32°C treatment (precision) and the minimum and maximum recorded temperatures at any point during the 32°C treatment were 30.0 and 32.9°C (reliability) (Fig. 2B). Similar results of accurate control of temperatures as imposed by the usage of IR heaters are reported in previous studies [12,17]. However, a previous study reported short episodes of tissue temperature increases up to 14 °C above set point [17]. We have not observed any "thermal shock" type rise in tissue temperature using the optimized conditions described here. The stability of the tissue temperature in the present study can be attributed to the combination of two factors: (1) the smooth, very rapid modulation of heater output provided through the combined use of the phase-angled fired SCR power controllers with the autotuned PID temperature controllers; which prevented thermal shock not only of the heating elements, but also of the target vegetation; and (2) the use of the fast response, low noise air-sensing thermocouples, which were subject to very little temperature buffering of the target and system heating response. A previous study had reported very little change in the surrounding air temperature, although canopy temperature differences were achieved [24]. A difference between the above mentioned study and other reported IR vegetation warming studies compared to the present study is our use of air temperature, instead of canopy temperature for controlling system response.

**Figure 3:**
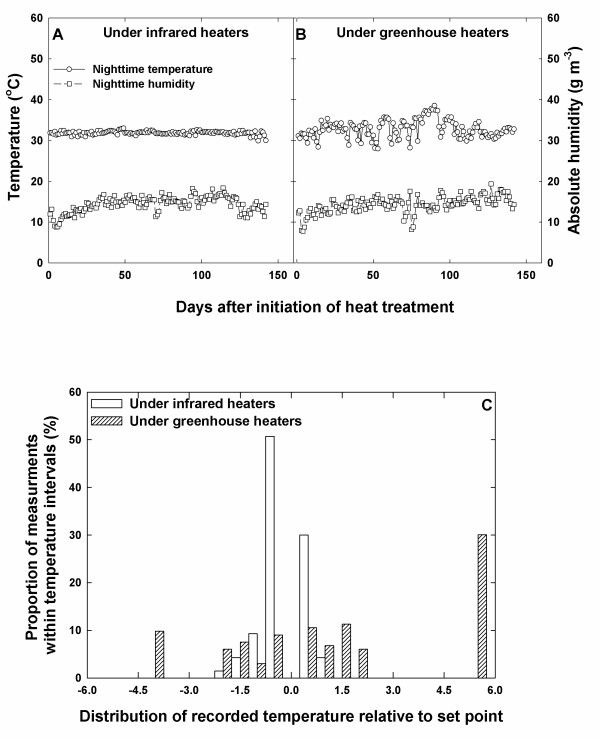
**Temperature and humidity under IR heating system and greenhouse conditions**. The night temperatures and humidities were monitored using standalone sensor/loggers (HOBOs) under IR heating system (A) and greenhouse conditions (B). The night temperature was set at 32°C. Distribution of recorded temperature to the set point for IR and greenhouse heating systems (C).

The IR heating system had more precision, accuracy and reliability in maintaining set point temperature compared to greenhouse heating (Fig. 3A, B, C). The paired t-test results indicated no differences between absolute humidity under IR and greenhouse heating (Fig. 3A, B). Moreover, the humidity during the cropping season was 14.3 and 14.4 gm m^-3 ^under the high night and ambient night temperature treatments, respectively, suggesting that the VPD was not altered to any unnatural extent through a change in absolute humidity during the IR heating. The ability to maintain the same absolute humidity in the presented study will provide flexibility in studying plant physiological response to elevated temperature in various ways, and was possibly due to the heating of unenclosed areas with light, but nearly constant, wind providing some mixing of the air.

**Figure 4:**
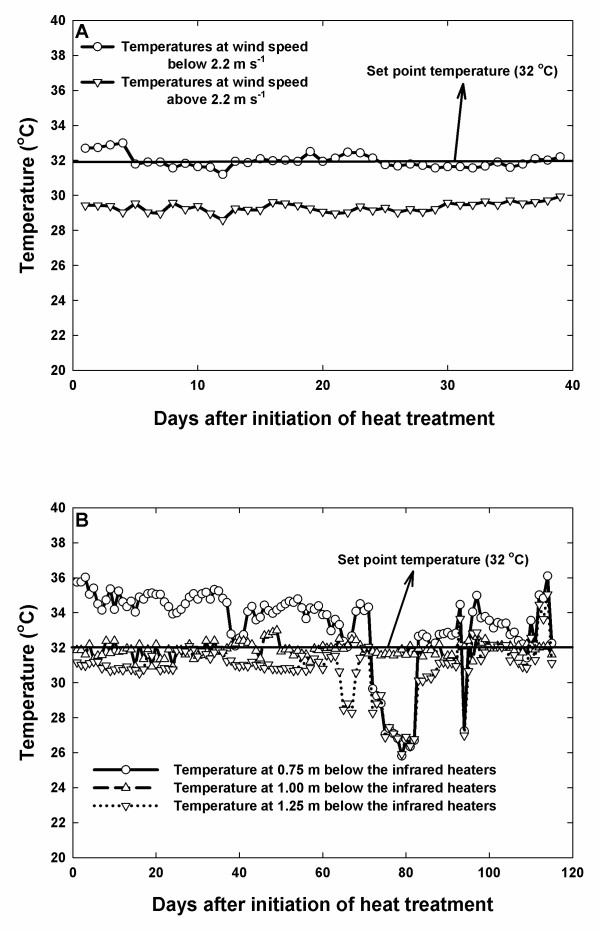
**Temperatures as affected by wind velocity and mounting height of the IR heaters**. To determine the ability of the IR heating system to control the temperatures, the IR heating lamps were exposed to wind velocities above and below 2.2 m s^-1 ^(A) and mounted at different heights (B).

The accuracy of the IR heater in maintaining the set point temperature greatly decreased with a wind velocity of 2.2 m s^-1 ^(Fig. 4A). At 2.2 m s^-1^, the IR heaters were off by 3°C. Similar results of decreased IR heater thermal radiation efficiency with increase in wind speed have been reported in previous studies [3,17]. However, the decrease in efficiency with wind speed can be adequately estimated [3]. In the presented setup, the IR heaters were able to maintain the set point temperatures when mounted 1 m above the canopy (Fig. 4B), however, further increasing the mounting distance above the canopy also decreased the ability of the IR heaters to maintain the set point temperatures. Similar results of decrease in the ability to maintain the set point temperatures with increase in mounting distance were reported by Kimball (2005) [3].

## Conclusion

The described IR heating system with the phase-angled-fired SCR power controller, autotuned PID temperature controllers, and fast response, low noise, air temperature thermocouples meets the utilitarian requirements of a heating system for plant physiology studies in that the elevated temperature can be accurately, precisely, and reliably controlled, and can be scaled in replicated study of populations of plants with minimal perturbation of other environmental factors. Changes to the physiology that can alter plant tolerance to abiotic stresses, such as "thermal shock" events or unusual alteration to the VPD due to change in the canopy to air temperature difference or change in the absolute humidity, are avoided. The combination of the lack of effect on other environmental factors and lack of unintended effects on the plant physiology indicate that the presented apparatus is specifically suitable for study of plant physiological response to high night temperature. The described IR heating system was able to maintain constant set point temperature, provided the heaters were not too high above the vegetation. Furthermore, wind speeds of or above 2.2 m s^-1 ^decreased the efficiency of this IR heating system. This IR heating system can be used in conductance of studies evaluating plant physiological response to high nighttime temperature.

## Competing interests

The authors declare that they have no competing interests.

## Authors' contributions

AM and LT conceived the project, designed experiments, and prepared the manuscript.

AM conducted the experiments and developed modifications to the instrumentation. LT designed the instrumentation and acquired funding. AM and LT read and approved the final manuscript.
